# Instinct of Appetite

**Published:** 1876-01

**Authors:** 


### INSTINCT OF APPETITE

Observant farmers know that one kind of grain, or seed, or plant, will flourish luxuriantly in a particular field, while another in that same field will grow feebly, and fail to arrive at perfection; it is because the soil in the former instance contains an element which nourishes the thriving plant, and in the latter case, it is deficient in that element which is the life of the sickly growth, and yet there is nothing amiss in the soil or in the seed—simply a want of adaptation. So in the case of a mother and her new-born child; both may be in ordinary good health, and yet the child dwindles and dies, not because there is essential disease in either, but because there is a want of mutual adaptation. In a few days there may be a change, and all is right. But it is interesting to remark the wisdom of Omnipotence in implanting an instinct for the child's safety, and it refuses to take the breast, or, if intense hunger impels it, it is done unwillingly, and Nature may, to some extent, be conquered, and the infant may come to tolerate, what it could not welcome, but it will die for all that.

Another parallel in agriculture is, that for a number of years a field will give abundant crops of a particular grain, but after a while they become less and less bountiful under the same culture, and finally, there is a total failure. In like manner, many of us have observed in our own persons, that for a long time we had a hearty relish for a particular kind of food, it almost seemed that we could never eat enough of it; but in process of time, the expression escapes us: "I don't care any thing about it now;" in some instances there is a positive aversion.

We constantly notice, at our own table, that a child will be ravenously fond of a particular dish, and after a while turns from it. The reason is, that there was a constituent in the much-loved food which the system required, and which it drank up greedily until it was fully supplied, and then instinct would receive no more. A thirsty man, like the arid soil, drinks in the water until the one is full and the other is saturated, and then the water is refused or rejected. The soil will not receive it, and it flows off; and when a man has enough, he becomes nauseated if he tries to drink more. To most persons, water has a very disagreeable taste, if it is attempted to be forced.

The practical conclusion to be drawn from these facts is simply this: Do not force your children or yourselves to take one single mouthful of any food or drink which they do not like. In sickness or health, consult the instincts of the appetite, and yield to them implicit and instant obedience. There is sometimes a morbid appetite, and if indulged in freely, injurious, if not fatal effects may follow; but in the most of these cases even, we prefer to believe that it is the quantity which does the harm, and not the quality: so that we are in the habit of saying to some classes of dyspeptics: "Eat what you most crave; but if you find that it is uniformly followed by some disagreeable feeling, instead of discarding that article of food, take half as much next time, and continue to diminish the quantity until it is found out how much of its favorite dish Nature can take with perfect impunity: if a spoonful only can be taken with perfect impunity, give Nature that spoonful as long as she craves it."

Most of us can call to mind cases where a craved dish or drink was most imperatively forbidden, under fear of death, if indulged in; and yet, the patient in desperation, has gotten up in the night, satisfied the appetite, and recovered from that hour. We advise the safer plan: take a very little at a time of what is so earnestly craved, and gradually feel the way along to an amount which Nature will bear. Physicians may rest assured that if the instincts of the invalid and the convalescent were more closely observed and studied, they would be more more successful, with less medicine.

---

TO-DAY, a gentleman expressed his willingness to give us a thousand dollars, to write him a book of a hundred pages, on HEALTH AND DISEASE. To make such an one, that would be safe, true, and practical, would require time and seclusion, neither of which do we hope to be ever able to command; for the shadows of the long night are even now beginning to extend themselves. But the pith of such a book may be comprised in a dozen words: HEALTH IS MAINTAINED BY SELF-DENIAL; DISEASE IS ENGENDERED BY SELF-INDULGENCE.

